# Comparison of the CDC Backpack aspirator and the Prokopack aspirator for sampling indoor- and outdoor-resting mosquitoes in southern Tanzania

**DOI:** 10.1186/1756-3305-4-124

**Published:** 2011-06-30

**Authors:** Marta F Maia, Ailie Robinson, Alex John, Joseph Mgando, Emmanuel Simfukwe, Sarah J Moore

**Affiliations:** 1 Disease Control Department, London School of Hygiene and Tropical Medicine, Keppel Street, London WC1E 7HT, UK; 2 Biomedical and Environmental Thematic Group, Ifakara Health Institute, Ifakara, Morogoro, Tanzania

## Abstract

**Background:**

Resting mosquitoes can easily be collected using an aspirating device. The most commonly used mechanical aspirator is the CDC Backpack aspirator. Recently, a simple, and low-cost aspirator called the Prokopack has been devised and proved to have comparable performance. The following study evaluates the Prokopack aspirator compared to the CDC backpack aspirator when sampling resting mosquitoes in rural Tanzania.

**Methods:**

Mosquitoes were sampled in- and outdoors of 48 typical rural African households using both aspirators. The aspirators were rotated between collectors and households in a randomized, Latin Square design. Outdoor collections were performed using artificial resting places (large barrel and car tyre), underneath the outdoor kitchen (*kibanda*) roof and from a drop-net. Data were analysed with generalized linear models.

**Results:**

The number of mosquitoes collected using the CDC Backpack and the Prokopack aspirator were not significantly different both in- and outdoors (indoors p = 0.735; large barrel p = 0.867; car tyre p = 0.418; kibanda p = 0.519). The Prokopack was superior for sampling of drop-nets due to its smaller size. The number mosquitoes collected per technician was more consistent when using the Prokopack aspirator. The Prokopack was more user-friendly: technicians preferred using the it over the CDC backpack aspirator as it weighs considerably less, retains its charge for longer and is easier to manoeuvre.

**Conclusions:**

The Prokopack proved in the field to be more advantageous than the CDC Backpack aspirator. It can be self assembled using simple, low-cost and easily attainable materials. This device is a useful tool for researchers or vector-control surveillance programs operating in rural Africa, as it is far simpler and quicker than traditional means of sampling resting mosquitoes. Further longitudinal evaluations of the Prokopack aspirator versus the gold standard pyrethrum spray catch for indoor resting catches are recommended.

## Background

There are numerous methods to sample adult mosquitoes, the suitable technique is dependant on the question to be answered. Most sampling methods aim at collecting mosquitoes in a particular physiological stage, e.g. host-seeking or ovipositing. Although these methods tend to provide large numbers of specimens through the use of attractants, they do not deliver representative samples of the mosquito population. Sampling of adult resting mosquitoes provides a cross-sectional sample of the whole population - giving more representative information on age structure, physiological condition and sex ratio of the population [1]. Mosquitoes can be found resting indoors or outdoors according to species-specific behaviour and season [2,3]. Outdoor-resting mosquitoes usually choose natural shelters such as tree hollows, animal borrows, broken logs, holes in the ground and vegetation [4]. Few mosquito species choose to rest inside human dwellings, but the ones who do are usually important disease vectors that are highly synanthropic [5-7]. In the context of disease monitoring and control, sampling of indoor-resting mosquitoes is an important efficacy indicator of household-level interventions as well as the degree of community protection that an intervention may provide[8-10]. However, as vector control is conducted throughout the African continent there is a possibility that a mass killing effect may create ecological niches for hitherto less relevant anophelines that subsequently may act as malaria vectors[11]. Indeed, an important exophilic *Anopheles gambiae *subgroup from West Africa was only recently identified due to the reliance on indoor sampling methods[12]. Thus it is important to develop standardised tools to monitor changes in mosquito populations both inside of human homes and in the immediate vicinity.

Indoor-resting collections may be performed using knock-down pyrethrum spray collections (PSC), oral aspirators or battery-powered aspirators [1]. The most widely used method is PSC because it is a well-established and standardised method that does not depend on individual ability or motivation to collect mosquitoes. Another advantage of the PSC is that it directly reduces the number of host seeking mosquitoes as well as other unwanted organisms, which is often appreciated by householders. However, if repeated sampling needs to be undertaken the presence of pyrethrum in the house will bias results by effecting subsequent mosquito house entry. Furthermore, the PSC method is cumbersome, time consuming and disturbing to the population because it requires removal of all furniture, food, water and animals from dwellings prior to spraying. This disturbance might encourage individuals to refuse permission for their houses to be sprayed on future occasions. In addition, African rural houses are difficult to completely seal off and once the pyrethrum is sprayed, mosquitoes may become irritated and leave the household before being knocked-down. Consequently, this effect might overestimate the proportion of blood fed mosquitoes inside households as heavy, engorged females have more sluggish flight and don't escape as easily as others [13]. Of greatest significance, it can only be used to sample endophillic mosquitoes that may only comprise a portion of all disease vectors in a given area.

Collection of resting mosquitoes using simple mouth aspirators has been used to successfully sample mosquitoes, but requires methodical and attentive work that is highly dependent on individual skill and motivation, and can be aggravating to the lungs [14]. Battery-powered aspirators reduce the level of skill and motivation needed by the operator due to their larger sampling radius and suction, and may therefore be used to deliver a more representative sample of mosquitoes especially those species that do not rest in buildings[15]. Historically, several types of electrical devices have been tested, including modified vacuum cleaners of different models and sizes[16-19]. Nowadays the most commonly used mosquito aspirator is the CDC Backpack aspirator, developed in the 1990's by the Centre for Disease Control and Prevention (CDC) [20,21]. Still, the CDC-BP has a few disadvantages: it weighs 12 Kg; it has a non-extendable suction hose, which hinders the collector from reaching higher ceilings or other sites of difficult access; it costs more than $470, and is only available from suppliers in the USA. Recently, Vazquez-Prokopec *et al*. [22] devised and tested in the field a simple, low-cost aspirator (40-75$), called the Prokopack. When tested in the sewers of Atlanta as well as indoor houses in Peru the Prokopack collected significantly more mosquitoes than the CDC-BP aspirator [22]. The accessibility to a low cost mosquito aspirator, which can be self made with simple material available in most areas of the African continent is needed to improve surveillance programs as well as facilitate researchers working in the field. However, the performance of different sampling tools can differ according to mosquito behaviour and the nature of structures to be sampled. Therefore, a time-limited comparison of the Prokopack and the CDC-BP aspirator was conducted in a rural African setting.

## Methods

### Study site

Experiments were conducted during the dry season of July 2010 in Mbingu village, 70 Km southwest of Ifakara, Tanzania. Mbingu lies in a 20 km wide flood plain towards the south of Kilombero valley at 8.21oS and 36.24oE. The site is characterized by typical rural houses surrounded by rice and banana fields close to the Londo River and the slopes of the Udzungwa Mountains. Malaria transmission was strongly holoendemic but is now moving to mesoendemic transmission as a result of malaria control activities [23].

### Villages

Houses within three small sub-villages within Mbingu were selected and enrolled in the project: Matete, Lower Sanje and Upper Sanje, with permission of the respective village leaders and individual household heads on written informed consent. Each community comprised 16, 14 and 18 households, respectively, totalling 48 households. Of those households, 38.5% had mud walls, 61.5% had burned mud brick walls; 61.7% were thatched with grass and 38.3% had corrugated steel roofs (Table 1). All households with exception of one in Lower Sanje owned and used bed-nets. The presence of livestock was also recorded as well as the number of household occupants.

**Table 1:** Characteristics of the households in Matete, Lower Sanje and Upper Sanje in Mbingu, Southern Tanzania

Village (number of households)	Number of occupants (%)			Household type (%)		Roof type (%)		Households with children under 5 years old (%)	Number of households using ITN's (%)	Number of households with livestock (%)		
	1-3	4-6	> 6	Mud walls	Burnt brick walls	Thatch roof	Corrugated iron sheets			Chickens	Goats	Pigs
Matete (16)	6 (37.5)	6 (37.5)	4 (25)	10 (62.5)	6 (37.5)	12 (75)	4 (25)	10 (63)	16 (100)	9 (56)	0 (0)	0 (0)
Lower Sanje (14)	5 (36)	8 (57)	1 (7)	2 (14)	12 (86)	6 (43)	8 (57)	11 (79)	13 (93)	12 (86)	0 (0)	1 (7)
Upper Sanje (18)	7 (39)	9 (50)	2 (11)	7 (39)	11 (61)	12 (67)	6 (33)	11 (61)	18 (100)	12 (67)	1 (6)	0 (0)

Numbers in parentheses are percentages of total number of households.

### Aspirators

**Figure 1:**
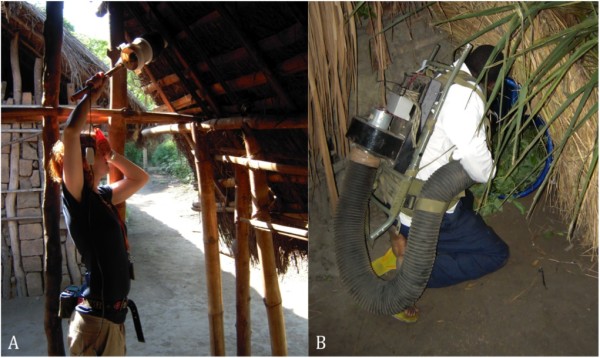
**Prokopack and CDC backpack aspirator**. A - Collector using the Prokopack aspirator to sample mosquitoes from underneath the roof of a *kibanda*. B - Collector using the CDC backpack aspirator to sample mosquitoes from inside a barrel.

The tested aspirators consisted of two CDC backpack aspirators model 1412 and two Prokopack aspirators (Figure 1). Each CDC-BP aspirator was connected to a 12 V wet-cell battery placed on the back of the collector. The Prokopack aspirator was operated on a 6 V dry-cell battery placed in a custom-made pouch and attached to a belt around the collector's waist. The batteries used were bought from a local hardware store.

### Mosquito collection

**Figure 2:**
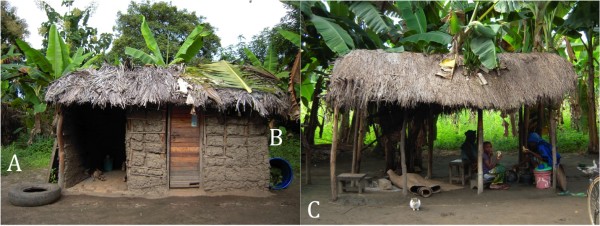
**Artificial resting shelters outside a participating household and typical Tanzanian outdoor sitting-area covered with a thatch-roof**. A - Car tyre; B - Barrel; C - *kibanda*.

**Figure 3:**
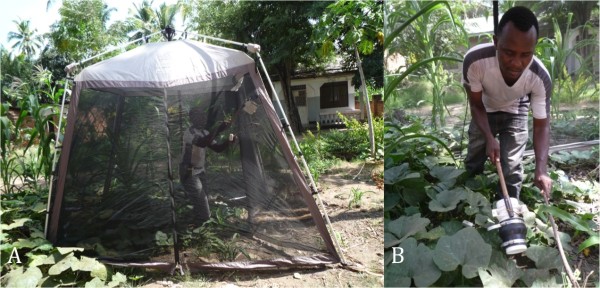
**Drop-net**. A - Field technician aspirating mosquitoes that landed on the walls of the drop-net with a Prokopack aspirator. B - Field technician beating slightly the vegetation to disturb the resting mosquitoes and aspirating them using a Prokopack aspirator.

A total of 16 experimental collections were completed over a period of 4 weeks during which the aspirators were rotated daily between four collectors. A total of 102 early-morning household collections were performed and each collector used each aspirator type on 14 occasions (n = 56). On average, each collector sampled two households per day, however on some occasions this was not possible as householders were not at home to grant permission to enter. Most households were sampled on two occasions. Aspirations were done in- and outdoors of all enrolled households starting at 06.00 hrs and finishing around 07:00. All technicians were previously trained by the same supervisor and had comparable aspiration techniques. They were spot-checked on random occasions throughout the collection to ensure their technique was correct. Walls and ceilings were systematically aspirated using progressive down- and upward movements along its entire length with a speed approximating 1 metre per second. Therefore, the time a collector spent aspirating was not pre-defined, but dependant on the size of the house that they were sampling. In addition to indoor collections, the following outdoor collections were performed in the immediate vicinity of each household:

- Large barrel - placed on its side and containing bits of cut vegetation (diameter = 0.65 m and depth = 0.75 m) (Figure 2a)

- Car tyre - (Figure 2b)

- Drop-net - floorless gazebo of approximately 13 m^3^ volume was dropped on the vegetation, which was then softly beaten with a stick to disturb the resting mosquitoes (Figure 3a and 3b).

- *Kibanda *- typical rural Tanzanian outdoor shelters where family members gather round to socialize, cook, eat and rest (Figure 2c). Resting mosquitoes were aspirated from underneath the thatch-roof.

Each type of outdoor collection was sampled 56 times using each aspirator model, with exception of the *kibanda*, as in some cases the households were renovating the thatch and the shelter was roofless. The *kibanda *was sampled 48 times using the CDC-BP and 45 times using the Prokopack aspirator. The statistical analysis accounted for unbalancing in the experimental design. Mosquitoes sampled from inside the drop-net were only collected using the Prokopack aspirator. This was due to operational difficulties using the CDC BP, which are discussed later.

### Mosquito identification

Sampled mosquitoes were identified to genus by a qualified technician. Anophelines of the A*n. gambiae *complex were submitted to PCR analysis in order to identify the species [24].

### Statistical analysis

Data were double entered and cleaned, and then coded before analysis to allow blinding of the individual performing the analysis. Statistical analysis was performed with STATA 11 (Stata Corp, USA). The data had a negative binomial distribution and was therefore analysed using generalized linear model with a negative binomial distribution [25] and a log link for generalized additive models where k of the negative binomial distribution is estimated from μ using maximum likelihood statistics [26]. The dependent variable was the total number of collected mosquitoes. The independent indicator variable was the aspirator type with CDC-BP assigned as the reference aspirator. Other independent variables included in the model were day, household, village cluster and collector. Household was included in the model as a random factor to account for multiple factors that could influence mosquito counts such as household occupancy, use of insecticide-treated nets or house structure. The influence that each independent variable had on the model was explored in a stepwise manner using Akaike's information criterion (AIC) to measure goodness of fit of the statistical model until AIC was optimized.

The interaction between collector and aspirator were analysed to determine which aspirator type is capable of delivering most consistent results, despite individual variability in collection ability. Data was fitted to a generalized linear model. In this analysis an interaction between collector and aspirator was used as the indicator variable and other independent variables were day and household. A randomly assigned collector was defined as reference to which all other collectors were compared. Again, the final model was derived in a stepwise fashion until the AIC was optimised.

### Ethical considerations

Ifakara Health Institute Institutional Review Board IHI/IRB/AMM/15-2010 and Tanzania National Institute of Medical Research NIMR/HQ/R8A/Vol-IX/780 granted ethical approval. Participation was upon written informed consent and only information on household structure was taken - no information on householders was requested. In the case where participants were illiterate, a member of technical staff or one of the householder's relatives read the consent form to them and a thumbprint taken. Consent could be withdrawn at any time.

## Results and Discussion

### Comparison of both aspirators

**Figure 4:**
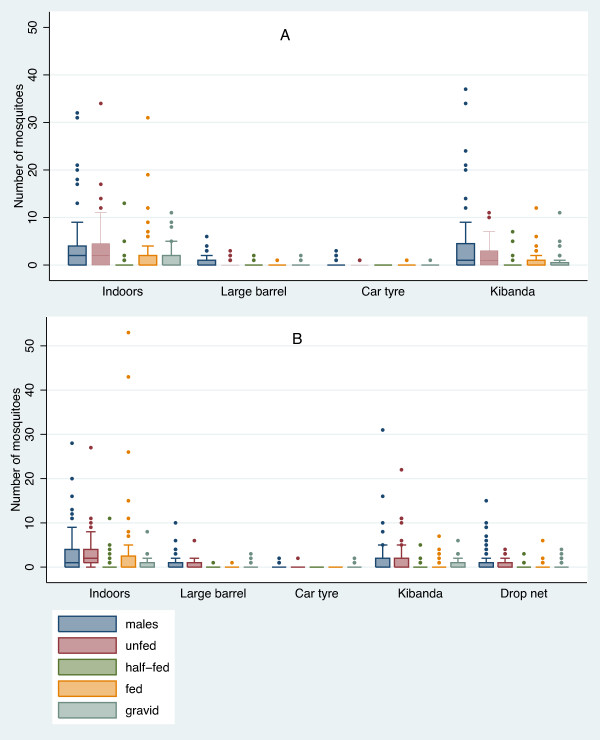
**Boxplots of the number of mosquitoes in different physiological stages collected indoor- and outdoor-resting**. A - Collections done using the CDC backpack aspirator indoors, and outdoors in artificial resting shelters: barrel and a car tyre, and underneath a *kibanda*. B - Collections done using the Prokopack aspirator indoors, and outdoors in artificial resting shelters: barrel, car tyre, inside a drop-net, and underneath a *kibanda*.

The CDC-BP aspirator and the Prokopack aspirator proved to be comparable in all sampling sites (Table 2). The number of indoor resting mosquitoes collected using each type of aspirator was not significantly different (p = 0.735), nor was the number of collected mosquitoes in the outdoor artificial shelters (large barrel p = 0.867; car tyre p = 0.418) or underneath the *kibanda *roof (p = 0.519). Also, the diversity of mosquito sex (Table 3), and physiological status (Figure 4a) collected using each aspirator were very similar for both aspirator types. *Anopheles gambiae *numbers were very low due to the climatic conditions when the study was carried out, but the method is consistent with the ratio of *An. gambiae *s.s. to *An. arabiensis *collected in the field site by human landing catch (Sangoro, unpublished).

**Table 2:** Comparison of total mosquitoes collected using the CDC-BP and the Prokopack aspirator in all sampling sites

Site	Aspiration method	n	N	IRR	Median	IQR	p-value
Indoors							
Indoor aspiration	CDC - BP	56	701	1	5	2 - 14.5	-
	Prokopack	56	658	0.93	6	3 - 15	0.735
Outdoors							
Large barrel	CDC	56	114	1	1	0 - 2.5	-
	Prokopack	56	101	1.04	1	0 - 2.5	0.867
Car tyre	CDC - BP	56	46	1	0	0 - 1	-
	Prokopack	56	31	0.78	0	0 - 1	0.418
Kibanda*	CDC - BP	48	420	1	4	1 - 8.5	-
	Prokopack	45	293	0.86	2	0 - 8	0.519

* fewer replicates of the kibanda were performed due to some roofs being under repair during the collection period

n - number of replicates; N - Total number of collected mosquitoes; IRR - incidence rate ratio; IQR - inter-quartile range.

**Table 3:** Total *Anopheles *spp., *Culex *spp., *Mansonia *spp. and *Aedes *spp. collected using CDC-BP and Prokopack aspirators

			*An gambiae *s. l. male**	*An gambiae *s.l. females**	*An gambiae *s.s males	*An gambiae *s.s. females	*An arabiensis *males	*An arabiensis *females	An funestus males	An. funestus females	*An coustani *males	An coustani females	*Culex *spp males	*Culex *spp females	*Mansonia *spp males	*Mansonia *spp females	*Aedes *spp. males	*Aedes *spp. females
															
		n			*An. gambiae *species*													
*CDC-BP*																		
*Indoors*	Indoor aspiration	56	2 (0.3)	2 (0.3)	2 (0.3)	1 (0.1)	0 (0)	1 (0.1)	0 (0)	0 (0)	0 (0)	0 (0)	256 (36.5)	425 (60.5)	0 (0)	16 (2.3)	0 (0)	0 (0)
*Outdoors*	Large barrel	56	0 (0)	3(2.6)	0 (0)	2 (1.8)	0 (0)	1 (0.9)	0 (0)	0 (0)	0 (0)	1 (0.9)	45 (39.5)	27 (23.7)	0 (0)	37 (32.5)	1 (0.9)	0 (0)
	Car tyre	56	1(2.2)	0(0)	0 (0)	0 (0)	0 (0)	0 (0)	0 (0)	0 (0)	0 (0)	0 (0)	12 (26.1)	6 (13)	0 (0)	27 (58.7)	0 (0)	0 (0)
	*Kibanda*	48	2 (0.5)	4 (1)	2 (0.5)	4 (1)	0 (0)	0 (0)	0 (0)	0 (0)	0 (0)	0 (0)	221 (52.6)	176 (41.9)	0 (0)	15 (3.6)	2 (0.5)	0 (0)
Subtotals			5 (0.4)	9 (0.7)	4 (0.3)	7 (0.5)	0 (0)	2 (0.2)	0 (0)	0 (0)	0 (0)	1 (0.1)	534 (41.7)	634 (49.5)	0 (0)	95 (7.4)	3 (0.2)	0 (0)
*Prokopack*																		
*Indoors*	Indoor aspiration	56	2 (0.3)	10 (1.5)	2 (0.3)	8 (1.2)	0 (0)	0 (0)	0 (0)	0 (0)	0 (0)	0 (0)	197 (29.9)	441 (67)	0 (0)	8 (1.2)	0 (0)	0 (0)
*Outdoors*	Large barrel	56	4 (3.8)	3 (2,9)	0 (0)	3 (2.9)	0 (0)	0 (0)	0 (0)	0 (0)	0 (0)	0 (0)	46 (44.2)	33 (31.7)	1 (1)	13 (12.5)	0 (0)	1 (1)
	Car tyre	56	0 (0)	1 (3.2)	0 (0)	0 (0)	0 (0)	1 (3.2)	0 (0)	0 (0)	0 (0)	0 (0)	11 (35.5)	6 (19.4)	0 (0)	13 (41.9)	0 (0)	0 (0)
	*Kibanda*	45	9 (3.1)	3 (1)	0 (0)	2 (0.7)	0 (0)	1 (0.3)	0 (0)	2 (0.7)	0 (0)	1 (0.3)	96 (32.8)	171 (58.4)	0 (0)	11 (3.8)	0 (0)	0 (0)
	Drop-net	112	0 (0)	0 (0)	0 (0)	0 (0)	0 (0)	0 (0)	0 (0)	0 (0)	0 (0)	1 (0.4)	138 (53.5)	99 (38.4)	3 (1.2)	17 (6.6)	0 (0)	0 (0)
Subtotals			15 (1.1)	17 (1.3)	2 (0.1)	13 (1.0)	0 (0)	2 (0.1)	0 (0)	2 (0.1)	0 (0)	2 (0.1)	488 (36.4)	750 (55.9)	4 (0.3)	62 (4.6)	0 (0)	1 (0.1)

**Totals**			**46 (1.8)**		**26 (1)**		**4 (0.2)**		**2 (0.1)**		**3 (0.1)**		**2406 (91.8)**		**161 (6.1)**		**4 (0.2)**	

* species composition: 87% *Anopheles gambiae *s.s and 13% *Anopheles arabiensis*. ** total amplified and non-amplified,

Numbers in parentheses are percentages of total mosquitoes collected with each aspirator in each sampling site.

However, the drop-net collections could not be performed with both aspirators because during preliminary experiments it was concluded that both the CDC backpack aspirator and the drop-net could not be carried in one trip between sites. Thus making two trips would have considerably shifted the time at which the households were sampled each morning and therefore compromise the results' comparability. In addition, the technicians were very unwilling to use the CDC-BP for the drop-net collections, as it was difficult to manoeuvre. Although the Prokopack allows the sampling of mosquitoes in higher ceilings this was not required during the trial as all houses enrolled in the project were typical rural Tanzanian houses with low ceilings.

### Interaction collector/aspirator

The Prokopack delivered more consistent results from different collectors than the CDC-BP aspirator (Table 4). Two of the collectors using the CDC-BP had significantly higher catches measured by incidence rate ration (IRR)(collector 1 IRR = 1; collector 2 IRR = 1.60, p = 0.18; collector 3 IRR = 3.19, p < 0.001: collector 4 IRR = 3.35, p < 0.001). Both collectors 3 and 4 sampled more than 3-fold the mosquitoes in comparison to collector 1. On the other hand, collectors using the Prokopack aspirator had more consistent results as only one of the collectors sampled a significantly lower number of mosquitoes (collector 1 IRR = 1; collector 2 IRR = 0.80, p = 0.528; collector 3 IRR = 0.72, p = 0.315; collector 4 IRR = 0.28, p < 0.001). Several collectors disliked the CDC-BP aspirator because of its bulkiness, weight and risk of acid burns due to leaking battery liquid. In rural areas of developing countries it is often difficult to find dry-cell car batteries so there was no alternative beside the wet cell battery. A 12V dry-cell battery can be directly ordered together with the CDC-BP, however these are quite costly (76US$ plus shipping from USA). Conversely, other collectors were very pleased to work with CDC-BP aspirator, as they perceived its large apparatus and bulkiness as a status symbol. All collectors were happy to work with the Prokopack aspirator as it was lighter to transport between collection sites, easier to manoeuvre and used a light 6V battery that was attachable to a belt pouch.

**Table 4:** Comparison of the number of mosquitoes collected by four technicians using CDC-BP and the Prokopack aspirator

	n	N	IRR	Median	IQR	p-value
*CDC - BP*						
Collector 1	53	163	1	2	(0 - 4)	-
Collector 2	54	200	1.60	0.5	(0 - 4)	0.18
Collector 3	58	475	3.19	2	(0 - 8)	< 0.001
Collector 4	51	454	3.35	2	(0 - 7)	< 0.001
*Prokopack*						
Collector 1	51	358	1	2	(0 - 7)	-
Collector 2	58	350	0.80	1.5	(0 - 5)	0.528
Collector 3	54	265	0.72	1	(0 - 6)	0.315
Collector 4	50	99	0.28	0.5	(0 - 4)	< 0.001

n - replicates; N - total mosquitoes (all sampling sites except drop-net); IRR - incidence rate ratio; IQR - Inter-quartile range

### Mosquito collections and comparison of sampling sites

Most of the mosquitoes were collected indoors (indoors aspiration N = 1359). The total number of mosquitoes collected outdoor collections was only slightly lower (∑outdoors N = 1263; large barrel N = 215; car tyre N = 77; *kibanda *N = 713; drop-net N = 258). The barrel outperformed the car tyre, which proved to catch very low number of mosquitoes. The drop-net also did not deliver high numbers of mosquitoes and was comparable to the barrel. Therefore in areas where outdoor structures such as kibandas are not available to sample the barrel is the preferable sampling tool because it is more consistent and convenient than the drop-net method.

The great majority of caught mosquitoes were males (N = 1049) and unfed females (N = 827). Most of the specimens were *Culex *spp. (N = 2406) followed by *Mansonia *spp. (N = 161) (Table 3). Very few anophelines were sampled (N = 46), the species encountered included *Anopheles gambiae *s.s., *An. arabiensis, An. funestus *s.l. and *An. coustani*. Collections were highest indoors; nonetheless outdoor collections were fairly high, particularly underneath the *kibanda *thatch-roof (Figure 4). Most of the blood-fed mosquitoes were found indoors (N = 371) and underneath the *kibanda *roof (N = 177). This suggests that under the *kibandas *outdoor transmission of vector-borne disease may take place as nearly everybody in rural Tanzania cooks and gathers in the evenings under *kibandas*.

## Conclusions

The Prokopack aspirator presents a valuable alternative to the CDC-BP aspirator as it delivers comparable results in addition to being easier to use especially over long periods of sampling. A great advantage of the Prokopack is that it does not need to be ordered from overseas, it can be self-assembled using easily attainable parts for a much smaller cost. Indeed, members of the team are now using units that were assembled from locally bought drainage pipe and computer fans. The battery on which it runs is also lighter, more affordable and easier to find in rural African settings. The Prokopack is a great tool for fieldwork in remote areas of sub-Saharan Africa, it is a valuable innovation for entomologists working in field research and may offer mosquito surveillance programs the possibility to expand their coverage, reduce their costs and therewith increase their efficiency. The study is limited because it was a short comparative evaluation of the relative merits of the Prokopack versus the CDC-BP aspirator. Longitudinal comparisons with pyrethrum spray catches (the gold standard method of indoor resting mosquito collection) with associated mosquito density measurements from Human Landing Catch are required to calibrate it against, in order to understand the merits of the Prokopack as a mosquito monitoring tool. However, we recommend that research of this nature is undertaken as the Prokopack may be used indoors and outdoors, is a cheap and effective tool that requires minimum skill to operate.

The findings of large numbers of blood-engorged mosquitoes outdoors in the *kibanda *indicates that vector-control programs within Tanzania should keep in mind the *kibanda *as a potential intervention site for the control of outdoor biting vectors. Programs that implement indoor residual spraying (IRS) in Tanzania may reach far better results by simply including the *kibanda *in their program. These household areas are ubiquitous in rural Tanzania. Families usually gather in evenings to cook, eat and socialize, most individuals only leave the *kibanda *to retire to bed after dark. The implications of this custom should be taken into consideration, especially since in some East-African regions it has been recorded that malaria vectors are shifting their behaviour to outdoor early-evening feeding [27-29]. There is a need for further research in order to better characterize the role of the *kibanda *in outdoor transmission of vector-borne diseases, most importantly malaria.

## Competing interests

The authors declare that they have no competing interests.

## Authors' contributions

MFM devised the experiment, drafted the manuscript, performed analysis and supervised the field experiments. SJM, assisted with the analysis and edited the manuscript. AR, ES and JM conducted the field experiments. AJ performed PCR analysis. All authors read and approved the final version of the manuscript.

## List of Abbreviations

AIC: Akaike's information criterion; BMFG: Bill and Melinda Gates Foundation; CDC: Centre for Disease Control and Prevention; CDC-BP: Centre for Disease Control Backpack; IQR: Inter-quartile range; IRR: Incidence rate ratio; PSC: Pyrethrum spray catch
